# Challenges and prospects in geriatric epilepsy treatment: the role of the blood–brain barrier in pharmacotherapy and drug delivery

**DOI:** 10.3389/fnagi.2024.1342366

**Published:** 2024-02-08

**Authors:** Xin Chen, Juan Luo, Min Song, Liang Pan, Zhichuang Qu, Bo Huang, Sixun Yu, Haifeng Shu

**Affiliations:** ^1^Department of Neurosurgery, Western Theater General Hospital, Chengdu, Sichuan, China; ^2^Department of Neurosurgery, The Affiliated Hospital of Southwest Medical University, Luzhou, Sichuan, China; ^3^Department of Pediatrics, Western Theater General Hospital, Chengdu, Sichuan, China; ^4^Department of Neurosurgery, Meishan City People's Hospital, Meishan, Sichuan, China; ^5^Department of Burn and Plastic, Western Theater General Hospital, Chengdu, Sichuan, China

**Keywords:** blood–brain barrier, geriatric epilepsy, antiepileptic therapy, antiepileptic drug, nanoparticle

## Abstract

The blood–brain barrier (BBB) is pivotal in maintaining neuronal physiology within the brain. This review delves into the alterations of the BBB specifically in the context of geriatric epilepsy. We examine how age-related changes in the BBB contribute to the pathogenesis of epilepsy in the elderly and present significant challenges in pharmacotherapy. Subsequently, we evaluate recent advancements in drug delivery methods targeting the BBB, as well as alternative approaches that could bypass the BBB’s restrictive nature. We particularly highlight the use of neurotropic viruses and various synthetic nanoparticles that have been investigated for delivering a range of antiepileptic drugs. Additionally, the advantage and limitation of these diverse delivery methods are discussed. Finally, we analyze the potential efficacy of different drug delivery approaches in the treatment of geriatric epilepsy, aiming to provide insights into more effective management of this condition in the elderly population.

## 1 Introduction

Geriatric epilepsy, characterized by an onset at or beyond the age of 65 years (Cloyd et al., 2006), presents a distinct epidemiological pattern. Epidemiological studies have delineated a bimodal distribution of epilepsy incidence, observing heightened rates in both young children and the elderly. Notably, the incidence of epilepsy escalates progressively post-50 years of age, reaching its zenith in individuals aged 75 and above (Hauser et al., 1993; Collaborators, 2019). In elderly patients, seizures are typically localized to specific brain regions, with convulsive symptoms being comparatively infrequent (Godfrey, 1989). This often leads to misdiagnoses of seizures as fainting or transient loss of consciousness in older adults (Ramsay et al., 2004). Such patients are at an increased risk of cognitive decline and psychiatric complications, factors that augment the likelihood of status epilepticus and related mortality (DeLorenzo et al., 1996; Martin et al., 2005).

Furthermore, geriatric populations exhibit heightened sensitivity to antiepileptic drugs, necessitating lower initial dosages and a more meticulous approach to medication regimen (Werhahn et al., 2011). Cerebrovascular disease is identified as the predominant cause of epilepsy in the elderly (Hauser et al., 1993), while blood–brain barrier (BBB) dysfunction emerges as a common pathological feature in this cohort (van Vliet and Marchi, 2022). Alterations in BBB functionality significantly influence drug delivery to the brain (Movahedpour et al., 2023), underscoring the need for a nuanced understanding of BBB dynamics in the context of elderly epilepsy treatment.

This review aims to elucidate the structure and function of the BBB and its role in the pathogenesis of geriatric epilepsy. It will also examine the impact of BBB on pharmacotherapy in elderly epileptic patients. Finally, the paper will explore innovative approaches to antiepileptic therapy, particularly strategies aimed at mitigating or circumventing BBB challenges, thereby offering fresh perspectives and strategies for treating geriatric epilepsy.

## 2 The structure and function of BBB

**Figure 1:**
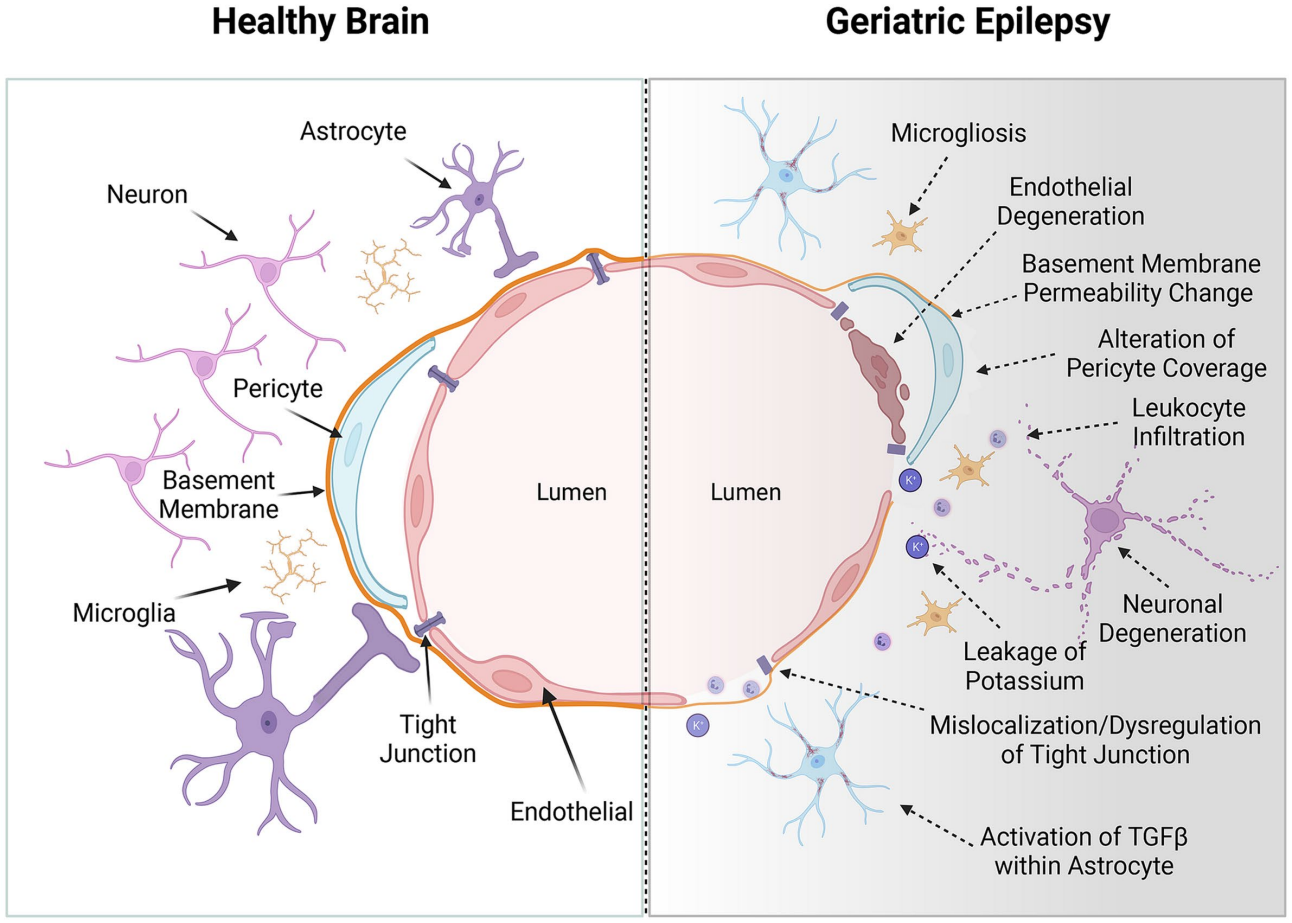
The blood–brain barrier (BBB) in the healthy brain and geriatric epilepsy patients’ brain. Left: In a healthy brain, the BBB consists of endothelial cells that are tightly connected through tight junction proteins, forming a primary physical barrier along the brain’s blood vessels. These cells are intricately surrounded by pericytes and perivascular macrophages embedded within the basement membrane. Additionally, the basement membrane is enveloped by the end feet of astrocytes. Furthermore, this unit contains inactive microglia and functional neurons, essential for the brain’s normal activation. Right: With advancing age, a convergence of adverse physiological processes can intersect with the pathogenesis of epilepsy. Age-related alterations include an increase in BBB permeability, leukocyte infiltration, pericellular shedding, mislocalization of tight junction, leakage of ions and activation of special signal pathway. Additionally, perivascular inflammation, along with the accumulation of amyloid and tau proteins, contributes to this complex scenario. These neuropathological changes, occurring concurrently, may collectively precipitate neurological disorders such as epilepsy and cognitive decline. The interplay of these factors underscores the multifaceted nature of age-associated neurological deterioration and highlights the need for targeted therapeutic strategies in the aging population. These alterations critically disrupt the central nervous system’s (CNS) delicate ‘excitation-inhibition’ balance, thereby potentially initiating or exacerbating the symptoms of epilepsy. Created with BioRender.com.

Comprehensive understanding of the BBB’s structure and the regulatory mechanisms of its permeability is paramount for developing diagnostic tools to assess BBB integrity and therapeutic interventions aimed at preserving or restoring normal BBB function in CNS diseases. The BBB is a critical selective barrier (Figure 1), constituted by brain endothelial cells, which regulates molecular exchanges between the bloodstream and neural tissue (Abbott et al., 2006). Its functionality hinges on the integrity of the neurovascular unit, comprising endothelial cells, pericytes, astrocytes, neurons, and the basement membrane (Sá-Pereira et al., 2012; Sweeney et al., 2016; Wang et al., 2021). Structurally, the BBB primarily consists of brain microvascular endothelial cells (BMECs) interconnected by tight junctions, adherens junctions, and gap junctions (Beard Jr et al., 2011; Spéder and Brand, 2014; Li et al., 2015). These intercellular junctional complexes significantly restrict the paracellular diffusion of solutes (Petty and Lo, 2002). Moreover, brain pericytes, astrocytic end-feet, neurons, and the extracellular matrix coalesce to form the neurovascular unit alongside BMECs, playing a pivotal role in inducing BBB properties during development and maintaining its integrity in adulthood (Kadry et al., 2020).

According to Yu et al. (2015), BMECs are distinguished from peripheral endothelial cells by their markedly low pinocytic activity and absence of fenestrations. The transcellular pathway across the BBB is intricately regulated by an array of membrane transporters and metabolic enzymes expressed in BMECs (Pottiez et al., 2009). Key efflux transporters such as P-glycoprotein (P-gp) and breast cancer resistance protein actively expel xenobiotics back into the bloodstream, whereas solute carrier transporters facilitate the influx of nutrients and ions into the brain (Kodaira et al., 2011). Additional membrane proteins and enzymes play crucial roles in controlling transcellular permeability (Raleigh et al., 2010; Del Vecchio et al., 2012; Sweeney et al., 2016). BMECs, in conjunction with the neurovascular unit, dynamically regulate BBB permeability to balance the metabolic demands of the central nervous system and protect against blood-borne toxins and pathogens (Daneman and Prat, 2015; Yan et al., 2021).

In various central nervous system disorders, including stroke (Bernardo-Castro et al., 2020), seizures (Uzüm et al., 2006), multiple sclerosis (Cramer et al., 2014), Alzheimer’s disease (Rosenberg, 2014), and brain tumors (Gerstner and Fine, 2007), BBB disruption is implicated, leading to increased permeability. This disruption facilitates the influx of potentially neurotoxic plasma components into the brain parenchyma (Keaney and Campbell, 2015). Diseases common in the elderly population, such as aging, hypertension, and diabetes, can further impair the integrity and functionality of the BBB (Zlokovic, 2008; Popescu et al., 2009; Prasad et al., 2014; Katsi et al., 2020).

## 3 Impact of aging on BBB alterations associated with the onset of geriatric epilepsy

Neuroimaging studies of the living human brain have revealed that disruptions in the BBB within the hippocampus are strongly linked to aging, a correlation that is particularly pronounced in patients with mild cognitive impairment (van de Haar et al., 2016; Sweeney et al., 2018; Nation et al., 2019). This phenomenon is corroborated by animal model studies, which have shown that the downregulation of tight junction proteins such as claudin-5 and occludin occurs with age, resulting in increased paracellular permeability of the BBB (Keaney and Campbell, 2015; Bors et al., 2018; Knox et al., 2022).

**Figure 2:**
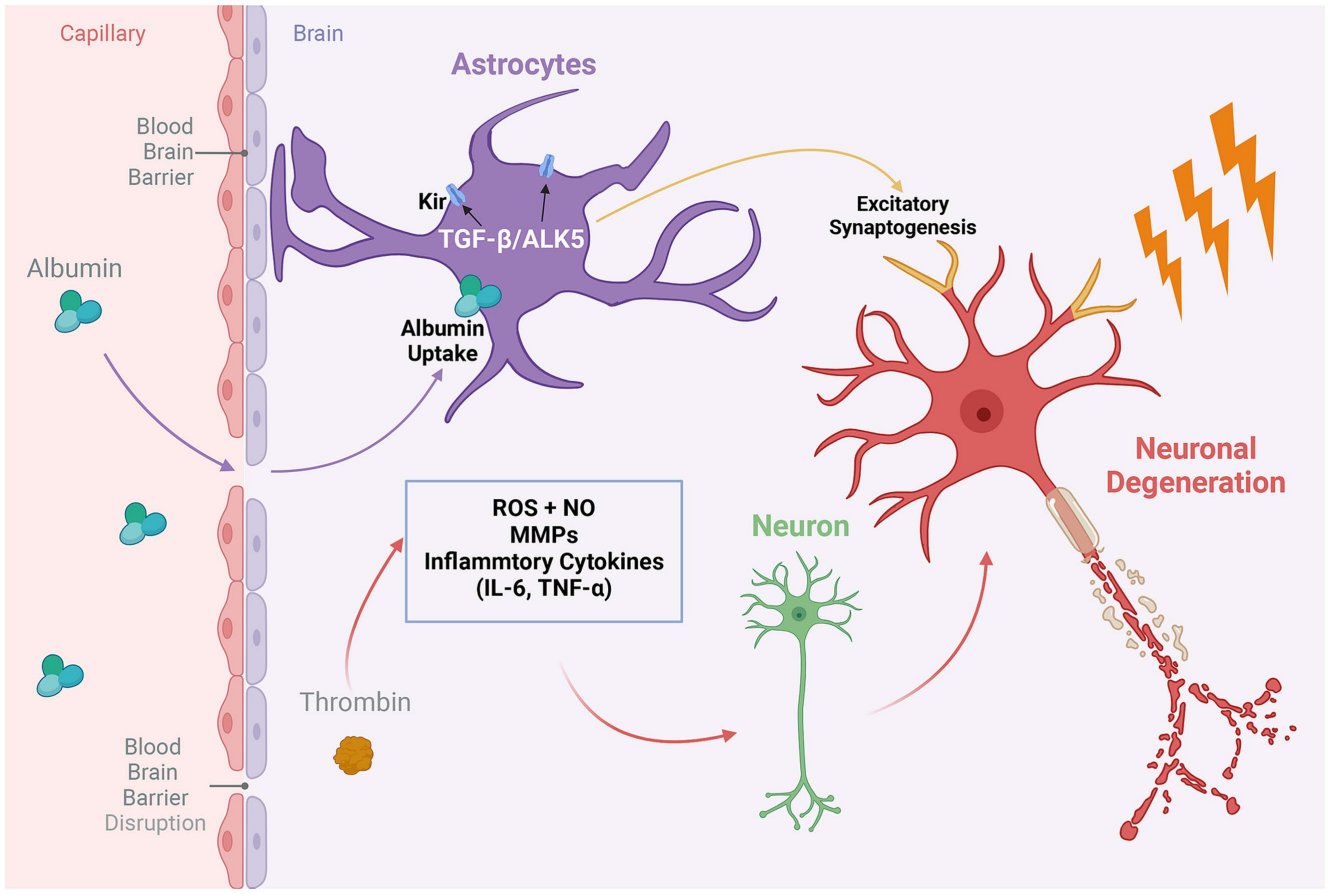
The role of proinflammatory proteins in the geriatric epilepsy brain. Thrombin, a key regulatory factor produced by endothelial cells during neuroinflammatory processes in the brain, impacts a variety of inflammatory mediators. These include inflammatory cytokines, matrix metalloproteinases (MMPs), reactive oxygen species (ROS), and nitric oxide (NO). The activity of these mediators can lead to the destruction of healthy neurons and precipitate neuronal degeneration, thereby exacerbating the neurological consequences of BBB disruption. On the other hand, compromised BBB integrity results in the leakage of serum albumin into the brain, subsequently triggering the activation of the TGFβ/ALK5 signaling pathway in astrocytes. This activation exerts a dual effect: it modulates the expression of potassium ion channels in astrocytes, affecting extracellular ion homeostasis, and concurrently promotes the formation of excitatory synapses in neurons. Together, these processes enhance neuronal excitability, potentially leading to epileptic discharges. Created with BioRender.com.

As part of the aging process, the BBB undergoes considerable structural and functional degradation (Figure 1). This degradation becomes evident when the BBB is compromised, manifesting in extensive alterations in comparison to a healthy state (Persidsky, 1999; Knox et al., 2022). Notable age-related changes in the BBB include significant modifications in cell-to-cell interactions, resulting in endothelial cell degeneration or atrophy and the reduced expression or mislocalization of tight junction proteins (Obermeier et al., 2013; Sweeney et al., 2019b). These alterations critically affect paracellular transport mechanisms (Erdő et al., 2017). Pathological conditions further lead to the upregulation of leukocyte adhesion molecules in endothelial cells, promoting leukocyte infiltration into the brain parenchyma and exacerbating oxidative stress through the release of reactive oxygen species, cytokines, and other mediators (Brochard et al., 2009; Gerwien et al., 2016; Profaci et al., 2020; Iannucci and Grammas, 2023; Figure 2). The heightened Reactive oxygen species consumption within the brain amplifies its vulnerability to oxidative stress (Segarra et al., 2021), with deleterious effects including oxidative damage to cellular components, activation of matrix metalloproteinases, and dysregulation of tight junction proteins and inflammatory mediators (Pun et al., 2009; Olmez and Ozyurt, 2012; Obermeier et al., 2013). These disruptions impair key cellular functions, including transport, energy production, and ion homeostasis (Olmez and Ozyurt, 2012). Additionally, the efficiency of P-gp in the BBB diminishes from middle age onwards, exacerbating the BBB’s transport dysfunction in conjunction with neuropathological burdens (Chiu et al., 2015; Banks et al., 2021).

At the cellular level, the process of aging is correlated with a notable reduction in the density and coverage of BMECs and pericytes. This reduction is particularly pronounced in brain regions that are more susceptible to age-related degeneration, such as the hippocampus (Montagne et al., 2015; Verheggen et al., 2020). Importantly, this decline associated with aging encompasses not just cellular density but also includes significant changes in the heterogeneity of astrocytes, oligodendrocytes, and microglia. These alterations collectively exert a profound impact on brain clearance mechanisms, myelination processes, and immune surveillance within the central nervous system (Kettenmann et al., 2011; Rodríguez-Arellano et al., 2016). Furthermore, changes in pericyte behavior, alongside alterations in glial cell function, are known to adversely affect BBB permeability and are implicated in the exacerbation of brain inflammation, further complicating the pathophysiological landscape (Rustenhoven et al., 2017; Sweeney et al., 2019a).

Consequently, this decline in BBB integrity and functionality has profound implications for neuronal health. BBB damage can precipitate epileptic seizures through various interconnected mechanisms. The compromised barrier allows for the leakage of substances such as potassium, albumin, or immune cells, which are intrinsically associated with abnormal neuronal firing (Seiffert et al., 2004; David et al., 2009; Fabene et al., 2010). Furthermore, BBB deterioration alters the balance of adenosine and glutamate transporters and catalytic enzymes, characterized by a decrease in adenosine and a elevation in glutamate levels, contributing to an increase in neuronal excitability (Grant et al., 2003; Parkinson et al., 2003). Additionally, BBB disruption can lead to a decrease in pH, negatively impacting the coupling of cerebral blood flow to neuronal activity and resulting in enhanced neuronal firing (Aaslid, 2006). These changes disrupt the central nervous system’s ‘excitation-inhibition’ balance, potentially initiating or intensifying epileptic symptoms.

Among the various pathways implicated in this disruption, the role of transforming growth factor beta (TGFβ)-associated pathway stands out as particularly significant in the progression of epilepsy, especially among the elderly (Figure 2). Research utilizing rodent models to emulate BBB leakage has elucidated that the infusion of serum albumin precipitates the activation of TGFβ signaling within astrocytes. This activation is implicated in engendering age-related neurological dysfunctions, brain aging phenotypes, heightened susceptibility to epilepsy, and cognitive impairments, as evidenced by several studies (Cacheaux et al., 2009; Milikovsky et al., 2019; Senatorov Jr et al., 2019). Itai et al. have highlighted the crucial role of the astrocyte ALK5/TGF-β pathway in mediating excitatory synaptogenesis post-BBB disruption, leading to seizures (Weissberg et al., 2015). Furthermore, the presence of albumin in the brain mediates the downregulation of potassium channels in astrocytes through TGFβ receptors, impacting potassium buffering and leading to increased extracellular potassium levels and neuronal overexcitability, thereby inducing epileptic discharges. The blockade of TGFβ receptors has been shown to significantly reduce the incidence of albumin-induced epilepsy (Ivens et al., 2007). Subsequent research revealed that serum-derived albumin preferentially activates the TGF-β receptor I activin receptor-like kinase 5 pathways in astrocytes. Notably, the TGF-β signaling inhibitor losartan effectively inhibits this pathway, preventing delayed recurrent spontaneous seizures, with effects lasting for weeks after drug discontinuation, highlighting its therapeutic potential (Bar-Klein et al., 2014).

## 4 Impact of BBB alterations on pharmacological challenges in geriatric epilepsy

In the management of epilepsy among elderly patients, antiepileptic drugs (AEDs) play a crucial role (Vu et al., 2018). Compared to their younger counterparts, older adults may derive greater benefits from AEDs but are simultaneously at an elevated risk for potential side effects (Hernández-Ronquillo et al., 2018). Consequently, in geriatric epilepsy treatment, the initial dosing and dosage increments of AEDs are typically reduced to half of those prescribed for younger individuals to enhance drug tolerance. It is often observed that elderly patients require only half the standard dose recommended for patients under 65 years of age (Sen et al., 2020). Moreover, the selection of appropriate AEDs for older adults is constrained by the increased likelihood of side effects and drug–drug interactions. For instance, traditional AEDs such as carbamazepine and phenytoin are often eschewed due to their adverse impacts on bone health, lipid metabolism, balance, and their propensity for enzyme induction (Sen et al., 2020). A meta-analysis has indicated that lamotrigine is better tolerated than carbamazepine in the elderly with epilepsy. Additionally, levetiracetam demonstrated a higher probability of seizure control compared to lamotrigine, with no significant difference in tolerability. Furthermore, no notable differences in efficacy and tolerability were observed between carbamazepine and levetiracetam (Lezaic et al., 2019). Another meta-analysis posited that lacosamide, lamotrigine, and levetiracetam are the most effective in terms of achieving seizure remission for old patients (Lattanzi et al., 2019). Patients with drug-resistant epilepsy, particularly older adults, may require an increased number of medications to control seizures and are more vulnerable to the neurotoxic effects of specific drugs (Trinka, 2003). This situation highlights the ongoing need for research into various drug delivery methods, including those involving the BBB.

The BBB defined by its intricate tight junctions and sophisticated efflux transport systems, is a critical physiological structure. Its role extends beyond the mere obstruction of drug delivery to the brain; it also regulates the distribution of therapeutic agents within brain tissues, presenting a significant challenge in treating central nervous system disorders (Cardoso et al., 2010; Li et al., 2011). Contemporary research has identified three protective mechanisms that drugs targeting the central nervous system encounter when interacting with the BBB (Angelow and Yu, 2007; Pardridge, 2007). The first is the enzymatic barrier, which restricts the entry of drugs and organic substances into capillary endothelial cells. The second is the cellular barrier, composed of contacts between BBB cells, where endothelial cells limit the passage of water-soluble substances and regulate vesicular transport and endocytosis. The third mechanism involves the ATP-binding cassette protein transport system, actively extruding certain drugs from the endothelial cells of brain blood vessels.

The BBB’s significance is particularly notable in epilepsy treatment, as it impedes the penetration of biotechnological drugs into the brain, thereby diminishing the effectiveness of antiepileptic medications (Zhao et al., 2020). In elderly epilepsy patients, a disrupted BBB structural integrity has been observed, which introduces complexities in traditional antiepileptic drug therapies. While it might seem intuitive that a compromised BBB would allow greater drug access to the brain, the reality is more nuanced. In fact, BBB leakage enables the entry of serum albumin into the brain, which adversely affects the brain distribution of many antiepileptic drugs that bind to plasma proteins, leading to suboptimal treatment outcomes (Marchi et al., 2009; Salar et al., 2014). Moreover, damage to the BBB can result in an increased expression of various drug transporters. This phenomenon contributes to pharmacoresistance in epilepsy, particularly due to the overexpression of ATP-binding cassette efflux transporters like P-gp and breast cancer resistance protein (van Vliet et al., 2014; Gonçalves et al., 2021). The excessive presence of these transporters restricts the entry of antiepileptic drugs into the brain, thereby playing a crucial role in the development of resistance to these drugs. Furthermore, oxidative stress, whether due to disease progression or AED side effects, may alter the expression of these BBB transport proteins (Grewal et al., 2017). Additionally, genetic variations and the increased expression of efflux transporter genes could be potential factors in pharmacoresistance. These transporters collaboratively act to inhibit antiepileptic drugs from delivering their therapeutic impact on the central nervous system (Soares et al., 2016).

Traditionally, one potential strategy to counteract the reduction in drug concentration within the central nervous system, caused by BBB disruption in elderly epilepsy patients, has been to increase the drug dosage. However, it is important to acknowledge that while most antiepileptic drugs can penetrate brain tissue, they also distribute to other organs, potentially leading to significant systemic toxicity (Perucca, 2002; Li and Ma, 2006). Such chronic toxicity, encompassing hematological disorders, hepatotoxicity, and other adverse effects, considerably constrains the clinical application of antiepileptic drugs (Perucca and Gilliam, 2012; Schmidt and Schachter, 2014). Consequently, developing targeted drug delivery systems specifically designed for the central nervous system may offer a promising approach to enhance drug efficacy in the brain while minimizing peripheral side effects. This strategy could prove invaluable in optimizing therapeutic outcomes in the treatment of epilepsy, particularly in the elderly.

## 5 Overcoming the BBB: advancements in targeted drug delivery for brain

**Figure 3:**
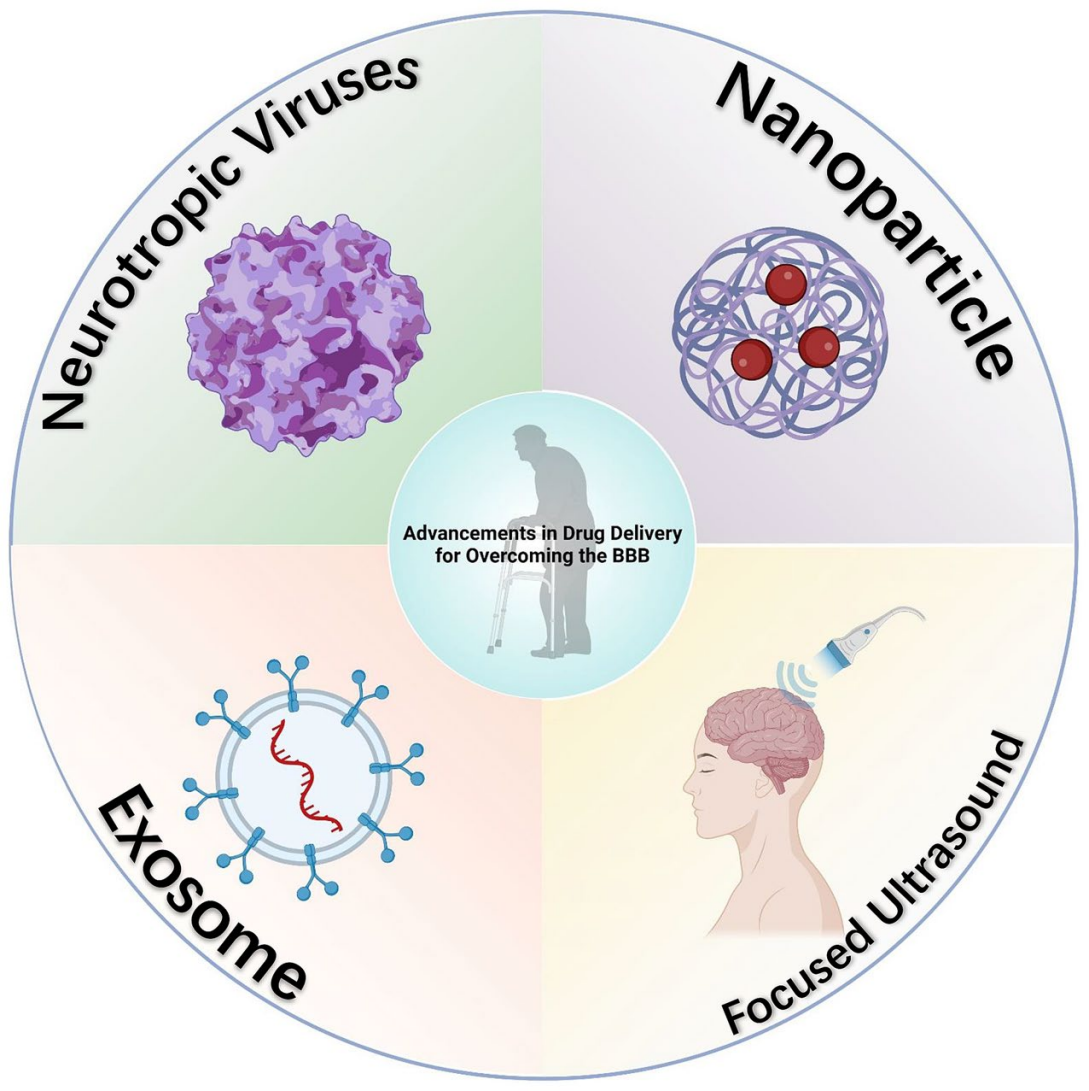
Diagram of advancements in drug delivery for overcoming the BBB. Several innovative methods for bypassing the BBB have been developed, and while not yet directly applied in the delivery of antiepileptic drugs, these approaches have demonstrated considerable potential in this area, as evidenced by existing research. Created with BioRender.com.

Contemporary approaches to drug administration across the BBB have been extensively reviewed in prior literature (van Vliet and Marchi, 2022), and thus, will not be reiterated here. In summary, the current therapeutic strategies for managing epilepsy in elderly patients predominantly encompass four mechanistic pathways: the modulation of pro- and anti-inflammatory balance (Fabene et al., 2008), the PDGF/TGF signaling pathways (Shen et al., 2019), pathways addressing oxidative stress (Luo et al., 2018), and the manipulation of matrix metalloproteinases (Broekaart et al., 2021). While these methodologies have shown potential in restoring cerebral vascular integrity and broadly controlling brain inflammation, their efficacy in epilepsy management and the efficiency of drug delivery via these mechanisms warrant further investigation. Given the unique physiological attributes of the BBB, there is a critical need for research focused on enhancing drug delivery efficiency through the BBB. Alternatively, exploring methods to directly bypass the BBB for the targeted delivery of antiepileptic drugs to the central nervous system is equally imperative. Here, we explore recent advancements in strategies designed to bypass the BBB for effective drug delivery to the central nervous system (Figure 3).

### 5.1 Neurotropic viruses

Neurotropic viruses exhibit a unique capacity to infiltrate the central nervous system via diverse mechanisms. These viruses inherently target the central nervous system, achieving entry by either directly crossing the BBB (Dahm et al., 2016) or utilizing immune cells as transport vehicles (Salinas et al., 2010). This attribute is critical for facilitating the delivery of therapeutic genes to brain cells (Körbelin et al., 2016; Powell et al., 2016), positioning neurotropic viruses as potential vectors for central nervous system targeted drug delivery.

In research focused on chronic temporal lobe epilepsy, the overexpression of neuropeptide Y (NPY) induced by recombinant adeno-associated virus (rAAV) vectors in epileptogenic regions has been shown to slow the progression of epileptic discharges. This approach has also been correlated with a notable reduction in the frequency of seizures (Noè et al., 2008; Noe et al., 2010). Moreover, the use of human foamy virus (HFV) as a gene transfer vector demonstrates efficiency in the expression of human glutamate decarboxylase (GAD) cDNA in hippocampal neurons. This expression leads to increased synthesis and release of gamma-aminobutyric acid (GABA) upon stimulation, thereby elevating the neuronal excitability threshold and contributing to an antiepileptic effect (Liu et al., 2005).

However, the clinical application of these findings faces significant challenges. Primary challenges involve addressing the immunogenicity (Rapti et al., 2012; Rabinowitz et al., 2019) and the inconsistencies in intracranial transduction efficiency (Foust et al., 2009; Gray et al., 2013) associated with these vectors. Additionally, the potential for inflammatory responses (Armangue et al., 2014; Ji et al., 2016) and toxicity underscores the need for rigorous evaluation and cautious application. Therefore, the optimization of viral vector designs is crucial. This entails minimizing immunogenicity and toxicity risks while maximizing targeting specificity to brain tissue. Such advancements are vital for ensuring the safe and effective utilization of neurotropic viral vectors in central nervous system drug delivery.

### 5.2 Nanoparticle

Nanoparticle (NP)-mediated drug delivery has emerged as a viable solution to the challenge of enhancing drug permeability across the BBB. Nanoparticles, defined as colloidal systems ranging from 1 to 1,000 nm in size, are adept at encapsulating therapeutic agents (Choradiya and Patil, 2021). This encapsulation not only improves the passage of drugs through the BBB but also enables precise targeting of specific brain regions affected by neurodegenerative and ischemic conditions (Solans et al., 2005). A diverse array of nanoparticles has demonstrated efficacy in brain delivery therapies. The effectiveness of these NPs is notably enhanced when their surfaces are modified with targeted moieties, thereby improving their specificity and efficiency in drug delivery (Farhoudi et al., 2022). In our investigation into the potential of nanoparticles to circumvent the BBB, we found their distinct advantages. These include their capacity to encapsulate diverse drug types, encompassing both hydrophilic and lipophilic compounds (Fonseca-Santos et al., 2015; Kamaly et al., 2016; DeMarino et al., 2017), and the potential for enhanced tissue-specific targeting through surface modifications (Saraiva et al., 2016). Furthermore, evidence suggests that nanoparticles can significantly increase the efficiency of drug delivery to the brain (Song et al., 2014; Maussang et al., 2016; Vilella et al., 2018), with exosome-based nanoparticles demonstrating enhanced stability in the bloodstream due to their resistance to phagocytic clearance (Yildiz et al., 2012; Hovlid et al., 2014).

Biodegradable nanoparticles have demonstrated considerable promise in augmenting the efficacy of antiepileptic drug therapies. Notably, surface functionalization of these nanoparticles has markedly improved their ability to permeate the BBB (Bonilla et al., 2022). For instance, polyssorbate-80 coated albumin nanoparticles have been effectively utilized to target the delivery of levetiracetam, a widely used antiepileptic drug, enhancing its brain-specific delivery and thus optimizing the treatment of epilepsy (Wilson et al., 2020). Additionally, the overexpression of P-gp is recognized as a pivotal contributor to the development of antiepileptic drug resistance (Löscher and Potschka, 2002; Löscher and Potschka, 2005; Luna-Tortós et al., 2008). To counteract this challenge, researchers have investigated co-embedding P-gp inhibitors and antiepileptic drugs in Pluronic P85-modified nanocarriers. This strategy has shown effectiveness in surmounting P-gp mediated drug resistance, particularly in the delivery of phenytoin (Fang et al., 2016). Furthermore, the innovative polypyrrole-poly-dopamine-phenytoin-Angiopep (PPY-PDA-PHT-ANG) nanoparticle system developed by Wu et al. represents a significant advancement in the field. This system employing copolymerization and surface functionalization techniques, not only achieves efficient drug loading and electro responsive release but also ensures precise brain-targeted delivery. This system distinguishes itself from traditional delivery methods through its capacity to respond to abnormal brain electrical activity, potentially contributing valuable insights to antiepileptic treatment strategies (Wu et al., 2022).

While conventional research continues to explore ways to exploit the physiological properties of the BBB for efficient brain drug delivery, some researchers have pursued an alternative approach through intranasal administration. This method directly bypasses the BBB, circumventing digestive tract absorption and first-pass liver metabolism, thereby enhancing drug bioavailability (Illum, 2003). Intranasal delivery can also target brain tissue directly, allowing for reduced dosages to achieve therapeutic effectiveness while minimizing systemic side effects (Pardeshi and Belgamwar, 2013). Its non-invasive nature and rapid efficacy make it particularly suitable for acute seizure management (Grassin-Delyle et al., 2012). Moreover, the potential for controlled release formulations extends its utility in chronic treatment, improving adherence, especially in patient groups with special needs, such as the elderly, where oral administration or intravenous injection may pose challenges (Illum, 2012).

In pre-clinical investigations, nanoparticles synthesized from polymers such as polylactic-polyethylene glycol copolymer and chitosan have demonstrated substantial promise for encapsulating antiepileptic drugs (Musumeci et al., 2018). These nanoparticle-based drug carriers have been notably effective in enhancing the transnasal cerebral delivery of drugs like carbamazepine and diazepam, significantly increasing their brain uptake (Sharma et al., 2015; Musumeci et al., 2018). Additionally, the incorporation of oxcarbazepine into nanoemulsions facilitated its transport through the nasal mucosa, resulting in an extended drug release profile and prolonged residence time in the brain (El-Zaafarany et al., 2016). In animal model trials for epilepsy treatment, these nanoparticle-encapsulated drugs exhibited superior antiepileptic efficacy compared to equivalent doses of the drugs in free form (Sharma et al., 2014; Musumeci et al., 2018). While these pre-clinical findings offer robust support for the utilization of nanocarriers in epilepsy treatment via the naso-brain route, comprehensive clinical studies are essential to validate their therapeutic effectiveness (Kapoor et al., 2016). Overall, the deployment of polymer-based nanoparticles for drug delivery through the naso-brain pathway emerges as a highly promising approach in the management of epilepsy.

Despite these promising attributes, there are notable limitations associated with nanoparticle technology. Their susceptibility to phagocytic engulfment and subsequent clearance from the circulatory system poses a significant hurdle (Hare et al., 2017). The intrinsic factors that influence drug release and targeting specificity further complicate their application. The necessity for brain-targeting ligands to enhance intracranial drug concentration introduces increased complexity and production costs (Saraiva et al., 2016). Moreover, the long-term stability of nanoparticles and potential issues of drug leakage warrant careful consideration (Dai et al., 2018). Future research endeavors should concentrate on optimizing critical attributes of nanoparticles, such as their stability, targeting accuracy, and drug loading capacity (Hare et al., 2017). The development of a safe, effective nanocarrier platform, and elucidating its interaction with disease mechanisms, are paramount for the successful integration of this technology in medical applications.

### 5.3 Beyond conventional methods

Numerous methods currently exist for circumventing the BBB, while not yet directly applied to the delivery of antiepileptic drugs, have shown significant potential in this domain according to existing studies. A notable example is the application of magnetic resonance guided low-intensity focused ultrasound (FUS). This technique has been evidenced to reversibly open the BBB, thereby enhancing the targeted delivery of drugs for brain therapies. In this field, Ali et al. have reported the findings of a preliminary clinical trial. This trial assessed the safety, feasibility, and reversibility of employing FUS techniques to breach the BBB in the hippocampus and entorhinal cortex areas (Rezai et al., 2020). Considering that these regions are focal points in epilepsy treatment, the deployment of FUS technology for the precise delivery of high-dose antiepileptic drugs offers substantial promise (Wiest and Beisteiner, 2019).

Moreover, exosomes represent another area of potential breakthrough. Owing to their endogenous nature, reduced toxicity, enhanced resistance to macrophage clearance, and extended half-life in the bloodstream, they are garnering significant interest. Research indicates that extracellular vesicles secreted by intranasally injected human bone marrow mesenchymal stem cells manifest robust anti-inflammatory properties in epileptic conditions. These exosomes are capable of reaching the hippocampal area within 6 h post-administration, effectively mitigating the loss of glutamatergic and GABAergic neurons associated with the epileptic state, and consequently, substantially diminishing hippocampal inflammation (Long et al., 2017). Despite the absence of studies on exosome-mediated antiepileptic drug delivery and the limitations of exosomes in drug payload capacity and efficiency, their advantages in safety, targeting specificity, and *in vivo* circulation duration position them as promising contenders to synthetic nanoparticles in the realm of antiepileptic drug delivery (de la Torre et al., 2020; Fu et al., 2020).

## 6 Conclusion and perspective

In summary, this review underscores the paramount importance of comprehending the dynamics of the BBB in managing epilepsy in the elderly. Although various innovative anti-epileptic strategies targeting the BBB have been discussed, their efficacy in elderly patients with epilepsy necessitates further exploration. With the rising prevalence of epilepsy in an aging population, unique pharmacological challenges emerge due to age-related alterations in the BBB. Future research endeavors should be directed towards understanding how these novel treatment strategies can be optimized in the context of such age-associated pathological changes. Given the intricacy of the BBB, advancements in targeted therapy and non-invasive drug delivery methods hold promise for enhancing treatment efficacy and, consequently, the quality of life for the elderly. The integration of precision medicine and emerging technologies in this field aims not only to ameliorate health outcomes but also to enrich our understanding of the aging nervous system. Adopting this approach will facilitate more personalized and compassionate care for our aging society, ultimately contributing to improved health management and enhanced quality of life for individuals with geriatric epilepsy.

## Author contributions

XC: Writing – original draft, Writing – review & editing. JL: Writing – review & editing, Investigation. MS: Writing – review & editing. LP: Writing – review & editing. ZQ: Data curation, Software, Writing – review & editing. BH: Conceptualization, Software, Writing – review & editing. SY: Writing – review & editing. HS: Writing – review & editing.
